# Serum Human Epididymis Protein 4 is Associated with Renal Function and Diabetic Kidney Disease in Patients with Type 2 Diabetes Mellitus

**DOI:** 10.1155/2019/4831459

**Published:** 2019-11-11

**Authors:** Miaomiao Zhang, Bing Zhao, Jing Xie, Yan Liang, Zaixing Yang

**Affiliations:** ^1^Department of Laboratory Medicine, Huangyan Hospital Affiliated to Wenzhou Medical University, Taizhou First People's Hospital, Wenzhou, Zhejiang, China; ^2^Department of Laboratory Diagnostics, Shanghai Changzheng Hospital, Shanghai, China

## Abstract

Human epididymis protein 4 (HE4) is an available tumor biomarker mainly for detecting ovarian cancer. However, it is unknown whether it can be a novel indicator for diagnosis of diabetic kidney disease (DKD). The aim of this study was to investigate the possibility of serum HE4 as a novel biomarker for DKD in patients with type 2 diabetes mellitus (T2DM). We enrolled 236 patients with T2DM and 82 healthy individuals. Serum HE4 was detected by ARCHITECT i2000 and compared between T2DM patients and healthy controls. The relationships between various variables and HE4 were analyzed by univariate or multivariate linear regression analyses. The receiver operating characteristic (ROC) curve was constructed to assess the diagnostic performance of HE4 for DKD. The association between HE4 and DKD was analyzed by logistic regression analysis. The serum HE4 level was significantly increased in T2DM patients (median, interquartile range (IQR), 69.7, 46.5–153.9, pM) compared with healthy control (median, IQR, 40.3 33.2–46.3, pM) (*P* < 0.001). Furthermore, it was higher in those with DKD (median, IQR, 211.1, 141.6–367.4, pM) than those without DKD (median, IQR, 55.5, 42.7–79.6, pM) (*P* < 0.001). The multivariable analysis showed that age, eGFR, HDL, CRP, and urea significantly independently correlated with HE4 level, while other variables did not. The ROC curve showed that the diagnostic performance of serum HE4 for DKD with 82.9 pM as the optimal cutoff value was good (AUC = 0.917, 95% CI: 0.872–0.961, *P* < 0.001, with a sensitivity and specificity of 92.1% and 76.9%, respectively) in T2DM patients. Multivariable logistic regression analysis showed that increased HE4 level was a significant, independent risk factor for DKD (OR, 95% CI, 57.7, 3.0–1112.9, *P* < 0.001) after adjusting for factors associated with HE4. Increased serum HE4 level is associated with decreased renal function and increased risks of DKD in patients with DM. It displays a good diagnostic value for DKD.

## 1. Introduction

The prevalence of diabetes mellitus (DM) has been rapidly increasing, due primarily to the increase in type 2 DM (T2DM), over the past few decades, which has made it an important public health issue [1]. Because of microvascular changes within the kidney, about 25–40% of DM patients may develop chronic kidney disease, an entity referred to as diabetic kidney disease (DKD) [2]. DKD that often leads to end-stage renal disease (ESRD) has been one of the most frequent complications of DM [3]. Furthermore, DKD is closely associated with increased risk of mortality in patients with DM [4]. Early diagnosis for DKD and timely nephroprotective therapy are helpful for preventing the progression of DKD toward ESRD and improving the prognosis. Although DKD is characterized by a distinct histopathological pattern, the thick needle biopsy is very rarely used because of invasiveness, nonspecific imaging, and lack of strict clinical indications in early stage of DKD. Therefore, it is needed to search for an early biomarker for DKD in DM patients.

Human epididymis protein 4 (HE4), firstly identified and characterized as a human epididymis-specific protein [5], nowadays, has been accepted as a useful biomarker for ovarian cancer or other malignancies [6, 7]. Recently, accumulating evidence demonstrated that serum HE4 level is influenced by renal filtration function and elevated in various renal diseases, such as lupus nephritis, chronic kidney disease (CKD), and acute kidney injury (AKI). [8–13]. However, it has remained unknown whether serum HE4 is elevated and associated with DKD in patients with T2DM. Accordingly, the aim of this study was to determine the serum HE4 level in DM patients, to identify the factors associated with HE4, and to investigate the possibility of serum HE4 as a novel biomarker for DKD.

## 2. Methods

### 2.1. Study Subjects

A total of 236 patients newly diagnosed as having T2DM (123 men and 113 women, age, mean ± SD, 61.6 ± 14.3 years) were recruited from Taizhou First People's Hospital between November 2016 and February 2019. All of those had never received any treatment of diabetes. The exclusion criteria included neoplasm, heart failure, chronic liver diseases, acute kidney injury, known renal diseases other than DKD, connective tissue diseases, urinary tract infection, pregnancy, gynaecological diseases, and so on. Of those, 63 (27%) patients had DKD that was defined by low estimated glomerular filtration rate (estimated glomerular filtration rate (eGFR) <60 mL/min/1.73 m^2^) for 3 months or more, or albuminuria (urinary albumin-to-creatinine ratio ≥30 mg/g or proteinuria >500 mg over a 24-hour period) in the setting of T2DM [14, 15]. A total of 82 healthy individuals (age, mean ± SD, 59.2 ± 11.6 years; female/male: 49/33) with no histories of DM were randomly recruited during the same period. Of the controls, the subjects were excluded if they had abnormal renal function, neoplasm, heart failure, or gynaecological diseases. This study was approved by local ethics committee (Number: 2019-KY003-01). The informed consent was not obtained from each subject, since we only analyzed medical database and the analysis did not have any influence on subsequent treatment.

Some clinical and demographic characteristics and laboratory data of the patients were obtained by medical record review. Body mass index (BMI) was calculated as weight/height^2^ (kg/m^2^). Hypertension was defined as systolic and diastolic blood pressure values over 140/90 mm Hg. Serum HE4 was determined by a two-step immunoassay chemiluminescence analyzer (Abbot ARCHITECT i2000, Abbot). The ARCHITECT HE4 assay had an imprecision of ≤10% total coefficient of variation (CV) and measurement range of 20.0–1500.0 pmol/L. Fasting blood glucose, serum triglyceridecreatinine (TG), total cholesterol (TC), low-density lipoprotein-cholesterol (LDL-C), high-density lipoprotein-cholesterol (HDL-C), creatinine, urea, uric acid (UA), C-reactive protein (CRP), urine creatinine, and albumin were measured by enzymatic assay (LAbOSPECT 008AS, HITACHI, Japan). Fasting serum C-peptide was measured by immunoassay (Maglumi 2000, Snibe Diagnostic, China). Fasting serum insulin was measured by immunoassay (UniCelDxiSOO ACCESS, BECKMAN COULTER, USA), Blood hemoglobin A1c (HbA1c) was determined in an automatic analyzer (G8-90SL, Tosoh corporation, Japan). eGFR was calculated by Chronic Kidney Disease Epidemiology Collaboration (CKD-EPI) equation [16]. The instruments and reagents used to detect aforementioned laboratory indicators have not been changed during the study period. The analyzers were routinely maintained according to the manufacturer's instruction. Dedicated reagents and standard methodologies were used. Two levels of quality controls were run every day. Internal quality controls (IQCs) were performed by Westgard alert rules. The total CV of both levels of quality control samples met the corresponding requirements. Additionally, we participate in the external quality assessment (EQA) for each aforementioned indicator, which is organized by National Center for Clinical Laboratories in China twice every year. The results of EQA indicated that our measurement quality is reliable and stable.

### 2.2. Statistical Analysis

Statistical analysis was performed by SPSS 17.0 software. Continuous variables were presented as mean ± standard deviation (SD) for normal distribution and median and interquartile ranges (IQR) for non-normal distribution. Categorical variables were described as frequencies and percentages. Continuous variables were compared using Student's *t* test or Mann–Whitney *U* test if appropriate. The *χ*2 test was used to compare categorical variables. When univariate linear regression analyses were conducted for assessing the correlation between HE4 and other variables, normally distributed data and log-transformed skew data were used. The variables with *P* < 0.05 were included in the multivariate linear regression analysis. Receiver operating characteristic (ROC) curve was plotted, and the area under the ROC curve (AUC) was calculated to evaluate the diagnostic performance of HE4. Univariate and multivariate logistic regression analyses were performed to analyze the association between HE4 and DKD. Results were considered statistically significant when *P* value was < 0.05.

## 3. Results

### 3.1. Baseline Characteristics of Study Population

**Figure 1:**
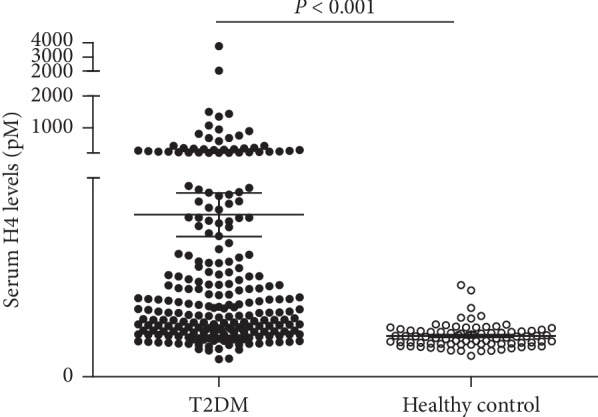
Serum HE4 levels in T2DM patients and healthy control. The results were presented as median and interquartile range (IQR). Statistical significance between the two groups was determined using a Mann–Whitney *U* test. HE4, human epididymis protein 4; T2DM, type 2 diabetes mellitus (*n* = 236); healthy control (*n* = 82).

**Figure 2:**
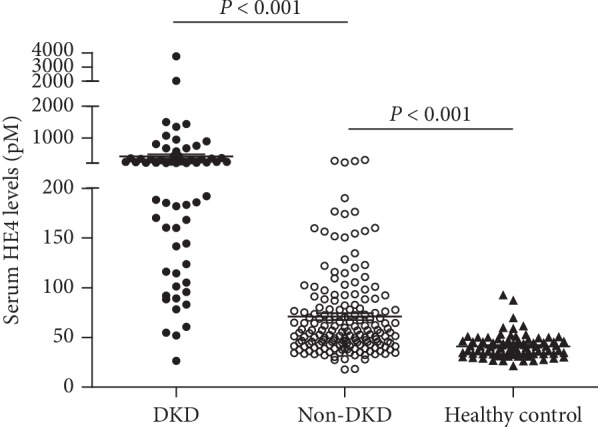
Serum HE4 levels in T2DM patients with or without DKD and healthy control. The results were presented as median and interquartile range (IQR). Statistical significance between groups was determined using a Mann–Whitney *U* test. DKD, diabetic kidney disease (*n* = 63); Non-DKD, nondiabetic kidney disease (*n* = 173); healthy control (*n* = 82).

There was no statistical difference for age (*P*=0.191) and sex (*P*=0.286) between T2DM patients and healthy controls. The HE4 levels were significantly increased in T2DM patients (median, IQR, 69.7, 46.5–153.9, pM) compared with healthy control (median, IQR, 40.3 33.2–46.3, pM) (*P* < 0.001) (Figure 1). Furthermore, the HE4 levels were higher in those with DKD (median, IQR, 211.1, 141.6–367.4, pM) than those without DKD (median, IQR, 55.5, 42.7–79.6, pM) (*P* < 0.001) (Figure 2).

T2DM patients were divided into two groups, below and above the HE4 median (69.7 pM) (Table 1). The patients with HE4 levels above the median were older and more likely to have hypertension, peripheral neuropathy, peripheral artery disease, and DKD (all *P* < 0.01). The levels of eGFR, serum C-peptide, CRP, urea, UA, and creatinin were significantly higher, but serum TC, HDL, and LDL were lower in the patients with HE4 levels above versus below the median (all *P* < 0.05).

### 3.2. Factors Associated with Serum HE4

Univariate linear regression analyses showed that age, hypertension, diabetic foot ulcers, blood glucose, C-peptide, urea, and uric acid were significantly positively, but eGFR, TC, HDL, and LDL negatively correlated with serum HE4. In the multivariable analysis, age, eGFR, HDL, CRP, and urea remained significantly associated with HE4 level, while other variables did not (Table 2).

### 3.3. Association between Serum HE4 and DKD

**Figure 3:**
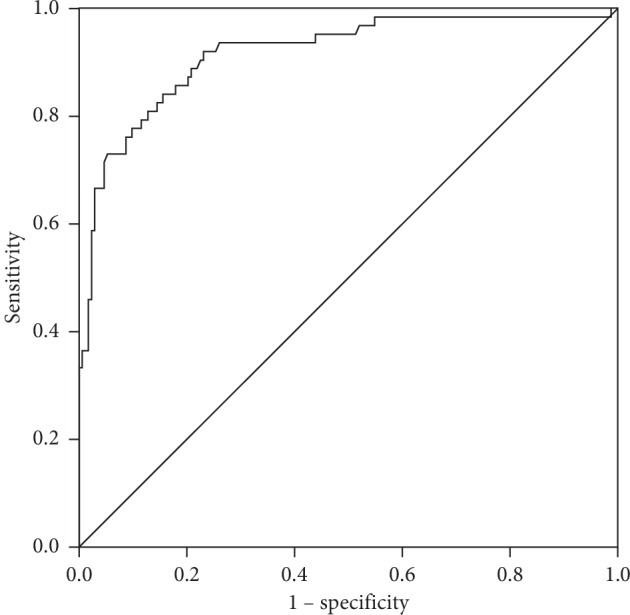
ROC curve of serum HE4 for the diagnosis of DKD in T2DM patients. The AUC value was 0.917 (95% CI: 0.872–0.961, *P* < 0.001) and the optimum cutoff value *f* was 82.9 pM. ROC, receiver operating characteristic; HE4, human epididymis protein 4; DKD, diabetic kidney disease; type 2 diabetes mellitus; AUC, area under the curve; CI, confidence interval.

The ROC curve showed that the diagnostic performance of serum HE4 for DKD with 82.9 pM as the optimal cutoff value was good (AUC = 0.917, 95% CI: 0.872–0.961, *P* < 0.001, with a sensitivity and specificity of 92.1% and 76.9%, respectively) in T2DM patients (Figure 3).

Univariate logistic regression analysis showed that T2DM patients with increased HE4 level per Log (pM) had a significant OR of 726.5 (95% CI, 128.8–4096.7, *P* < 0.001). Moreover, HE4 remained a significant risk factor for DKD (OR, 95% CI, 57.7, 3.0–1112.9, *P* < 0.001) after adjusting for factors associated with HE4 including age, HDL-C, CRP, and urea (Table 3).

## 4. Discussion

To the best of our knowledge, this is the first study to investigate the clinical significance of HE4 in T2DM. In this study, we found that serum HE4 level is increased in DM, especially in DKD. Furthermore, age, serum urea, and CRP are positively associated, but eGFR and HDL negatively, with the elevation of HE4. More importantly, serum HE4 is strongly associated with increased risk of DKD and can be used as a novel biomarker for DKD diagnosis in T2DM patients.

Several studies reported increased serum HE4 that is related to the decreased renal function in CKD or AKI patients [10–13]. Our previous studies found a similar result in SLE patients [8, 9]. Furthermore, increased HE4 may be a valuable predictor for lupus nephritis (LN) development in SLE patient without LN [9]. In the present study, we also found that serum HE4 showed a strong, negative correlation with eGFR in T2DM patients, even though adjusting for various confounding factors such as age, urea, uric acid, blood glucose, and blood fat. Therefore, we presumed that increased serum HE4 may be a reliable indicator to reflect renal dysfunction regardless of cause.

Furthermore, our current study demonstrates a strong association between increased serum HE4 and DKD, even after adjusting for other HE4-related factors. Increased serum HE4 level per tenfold may increase a 57.7-fold risk of DKD development in T2DM patients. Moreover, from the AUC analysis, we can conclude that serum HE4 is a good indicator for DKD diagnosis. Currently, eGFR and albuminuria are the best indicators for DKD screening, although neither is sensitive for early diabetic renal damage [17]. Because the diagnosis of DKD was based on eGFR and albuminuria in the present study, we cannot compare the diagnostic performance of HE4 with that of eGFR and albuminuria for early DKD. Anyways, our study suggests that it deserves an expectation whether HE4 can have a complementary role for eGFR and albuminuria in early diagnosis of DKD.

In addition, we identified some independent factors related to HE4 increase with the exception of eGFR in T2DM patients. Firstly, we found that age is positively correlated with serum HE4, which has also been found in chronic heart failure [18], systemic lupus erythematosus [8], and healthy individuals [19]. These results suggest that age should be considered when the clinical value of HE4 is investigated. Secondly, there is a positive relationship between serum HE4 and CRP, suggesting that HE4 may also be an inflammatory marker in T2DM. Similarly, the previous studies have found that serum HE4 may be an inflammatory marker in patients with SLE [8, 9] and cystic fibrosis [20]. Therefore, HE4 may play a role in the inflammatory process of DKD, although the detailed mechanisms remained unknown. Finally, serum HE4 shows a negative correlation with HDL-C, the decrease of which has been reported to be an independent risk factor for DKD development [21]. Although we cannot explain the reason for the close association, it is possible that interaction between HE4 and HDL-C may be involved the development and progression of DKD in T2DM patients.

There are some limitations that should be addressed. Firstly, since very few subjects received kidney biopsy, we could only select randomly the patients with DKD, the diagnosis of which was based on eGFR and albuminuria. Therefore, we cannot prove whether serum HE4 is a sensitive biomarker for early DKD based on kidney histological characteristics, especially compared with eGFR and albuminuria. Secondly, this is a single-center study with a limited power to evaluate the relationship between serum HE4 and DKD. Therefore, our current results should be interpreted with caution and be further confirmed by studies with larger size. Thirdly, this is a cross-sectional study that cannot confirm a causative relationship. Some prospective studies are needed to establish the causation between HE4 and DKD development.

## 5. Conclusions

In conclusion, increased serum HE4 level is associated with decreased renal function and increased risks of DKD in patients with T2DM. It displays a good diagnostic value for DKD. Since serum HE4 has widely been used as a tumor marker in clinical practice, it may be convenient and easy for this indicator to be used in DKD screening.

## Figures and Tables

**Table 1:** Baseline characteristics of T2DM cohort.

	Total (*n* = 236)	Below-median HE4 (*n* = 118)	Above-median HE4 (*n* = 118)	*P* value
Age (y)	61.6 ± 14.3	55.1 ± 11.9	68.0 ± 13.7	<0.001
Female, *n*	113 (48%)	59 (50%)	54 (46%)	0.515
BMI	23.8 ± 2.8	24.0 ± 2.9	23.6 ± 2.8	0.284
Current smoker, *n*	38 (16%)	18 (15%)	20 (17%)	0.859
Current drinker, *n*	32 (14%)	12 (10%)	20 (17%)	0.105
Hypertension, *n*	115 (49)	43 (36%)	72 (61%)	<0.001
Coronary heart disease	14 (6)	7 (6)	7 (6)	0.783
Peripheral neuropathy	143 (61%)	61 (52%)	82 (69%)	0.008
Peripheral artery disease	67 (28%)	24 (20%)	43 (36%)	0.009
Diabetic retinopathy	26 (11%)	11 (9%)	15 (13%)	0.533
Diabetic foot ulcers	16 (7%)	5 (4%)	11 (9%)	0.120
Diabetic ketosis	26 (11%)	15 (12%)	11 (9%)	0.533
DKD	63 (53%)	4 (5%)	59 (50%)	<0.001
Laboratory measurements				
eGFR	94.9 (66.1–115.6)	106.7 (95.0–128.4)	66.8 (45.1–94.7)	<0.001
Blood glucose	8.9 (6.3–12.9)	9.3 (6.8–13.2)	8.2 (5.8–12.1)	0.134
HbA1c	9.3 (7.8–11.2)	9.4 (7.6–11.2)	9.1 (7.9–11.3)	1.000
Insulin	9.5 (5.4–14.6)	9.3 (5.3–13.6)	10.4 (6.1–15.3)	0.287
C-peptide	1.9 (0.9–2.7)	1.8 (0.7–2.5)	2.1 (1.2–3.1)	0.007
TG	1.5 (1.1–2.3)	1.6 (1.1–2.5)	1.5 (1.2–2.1)	0.406
TC	4.4 (3.7–5.3)	4.7 (3.9–5.6)	4.2 (3.6–5.3)	0.029
HDL-C	1.1 (0.9–1.3)	1.1 (0.9–1.4)	1.0 (0.9–1.2)	0.007
LDL-C	2.4 (1.8–3.1)	2.6 (1.9–3.2)	2.2 (1.7–3.0)	0.037
CRP	3.2 (1.4–11.7)	1.9 (1.1–4.6)	5.6 (2.3–22.6)	<0.001
Urea	5.5 (4.4–7.5)	4.9 (4.2–5.7)	7.2 (5.3–10.6)	<0.001
Creatinine	66.0 (55.0–89.8)	58.0 (50.0–70.0)	83.5 (62.0–125.3)	<0.001
UA	312.0 (251.0–391.0)	282.0 (233.0–342.0)	360.0 (275.5–439.5)	<0.001

T2DM, type 2 diabetes mellitus; HE4, human epididymis protein 4; BMI, body mass index; DKD, diabetic kidney disease; eGFR, estimated glomerular filtration rate; HbA1c, hemoglobin A1c; TG, triglyceridecreatinine; TC, total cholesterol; HDL-C, high-density lipoprotein-cholesterol; LDL-C, low-density lipoprotein-cholesterol; CRP, C-reactive protein; UA, uric acid.

**Table 2:** Relationship between HE4 Levels and baseline characteristics in T2DM patients.

	Univariable			Multivariable		
	S*β*	*R* ^2^	*P* value	*β* ± SE	S*β*	*P* value
Age (*y*)	0.375	0.141	<0.001	0.005 ± 0.001	0.180	<0.001
Female, *n* (%)	−0.093	0.009	0.158			
BMI	0.044	0.002	0.501			
Current smoker, *n* (%)	0.038	0.001	0.557			
Current drinker, *n* (%)	0.103	0.011	0.115			
Hypertension, *n* (%)	0.233	0.054	<0.001	−0.032 ± 0.030	−0.046	0.292
Coronary heart disease	−0.054	0.003	0.406			
Peripheral neuropathy	0.073	0.005	0.266			
Peripheral artery disease	0.077	0.006	0.238			
Diabetic retinopathy	0.085	0.007	0.193			
Diabetic foot ulcers	0.208	0.043	0.001	−0.055 ± 0.059	−0.040	0.352
Diabetic ketosis	−0.102	0.010	0.119			
Laboratory measurements						
eGFR	−0.830	0.690	<0.001	−1.057 ± 0.104	−0.646	<0.001
Blood glucose	−0.255	0.065	<0.001	0.037 ± 0.072	0.022	0.607
HbA1c	−0.059	0.003	0.421			
Insulin	0.007	<0.001	0.917			
C-peptide	0.170	0.029	0.014	−0.057 ± 0.032	−0.077	0.075
TG	−0.089	0.008	0.177			
TC	−0.188	0.035	0.004	−0.347 ± 0.245	−0.114	0.174
HDL-C	−0.249	0.062	<0.001	−0.480 ± 0.142	−0.149	0.001
LDL-C	−0.163	0.026	0.014	0.055 ± 0.153	0.028	0.719
CRP	0.321	0.103	<0.001	0.083 ± 0.023	0.155	<0.001
Serum urea	0.716	0.513	<0.001	0.286 ± 0.112	0.153	0.011
Serum uric acid	0.402	0.162	<0.001	−0.140 ± 0.123	−0.054	0.256

T2DM, type 2 diabetes mellitus; HE4, human epididymis protein 4; BMI, body mass index; DKD, diabetic kidney disease; eGFR, estimated glomerular filtration rate; HbA1c, hemoglobin A1c; TG, triglyceridecreatinine; TC, total cholesterol; HDL-C, high-density lipoprotein-cholesterol; LDL-C, low-density lipoprotein-cholesterol; CRP, C-reactive protein; UA, uric acid.

**Table 3:** Association between serum HE4 and DKD in T2DM patients.

	OR	95% CI	*P* value
Unadjusted model	726.5	128.8–4096.7	<0.001
Adjusted model	57.7	3.0–1112.9	0.007

T2DM, type 2 diabetes mellitus; HE4, human epididymis protein 4; DKD, diabetic kidney disease; OR, odd ratio; CI, confidence interval. Adjusted model included factors associated with HE4, i.e., age, HDL-C, CRP, and urea.

## Data Availability

The original data used to support the findings of this study will be provided upon request.
